# Brassinin Abundant in Brassicaceae Suppresses Melanogenesis through Dual Mechanisms of Tyrosinase Inhibition

**DOI:** 10.3390/foods12010121

**Published:** 2022-12-26

**Authors:** Min-Kyeong Lee, Heeyeon Ryu, Hyeon Hak Jeong, Bonggi Lee

**Affiliations:** 1 Department of Food Science and Nutrition, Pukyong National University, Busan 48513, Republic of Korea; 2 Department of Smart Green Technology Engineering, Pukyong National University, Busan 48513, Republic of Korea

**Keywords:** brassinin, melanogenesis, tyrosinase

## Abstract

Brassinin is a phytoalexin abundant in plants, especially in cabbage, and has been reported to act as an anti-cancer and anti-inflammatory agent. However, limited studies are available to elucidate the functionalities of brassinin. Here, we tested the effects of brassinin on melanogenesis using cell-free and cell-based biochemical analysis and docking simulation. Cell-free experiments exhibited that brassinin has antioxidant and anti-tyrosinase activities. When applied to B16F10 cells stimulated with a melanogenesis inducer α-MSH, brassinin pretreatment significantly reduced melanin accumulation and cellular tyrosinase activity. Docking simulation indicates that the docking score of brassinin to the binding pocket of tyrosinase is better than that of kojic acid or arbutin, anti-melanogenic positive controls, indicating that brassinin inhibits melanogenesis at least partially by binding to and inactivating tyrosinase. In addition, qPCR results showed that brassinin reduced tyrosinase mRNA levels. Together, these results suggest that brassinin exerts anti-melanogenesis effects by inhibiting both the activity and mRNA expression levels of tyrosinase. Therefore, our study showed that brassinin has the potential to be used in pharmaceutical or cosmetic products for depigmentation.

## 1. Introduction

Melanin determines the color of mammalian hair, eyes, and skin and is responsible for protecting the skin against the harmful effects of ultraviolet (UV) radiation [1,2]. However, the overproduction or abnormal distribution of melanin is closely associated with hyperpigmentation-related skin diseases, including freckles, melasma, and solar lentigo [3,4,5]. Abnormal skin color changes may damage the appearance and cause emotional distress, ultimately reducing the quality of life. Thus, skin whitening agents are receiving great attention in the cosmetic and pharmaceutical industries due to their preventive effects on abnormal melanogenesis [6].

Melanogenesis occurs in melanosomes, the organelles of melanocytes, under the regulation of several melanocyte-specific enzymes, including tyrosinase, tyrosinase-related protein (TRP)-1, and TRP-2 [7,8,9,10]. Tyrosinase is a multifunctional copper-containing oxidase present in various animals, plants, and microorganisms that catalyzes the production of melanin by hydroxylating L-tyrosine to form 3,4-dihydroxyphenylalanine (L-DOPA) and oxidizing L-DOPA to dopaquinone [11,12,13,14]. Tyrosinase is a rate-limiting enzyme that plays an important role in the initial step of the melanogenesis pathway, and the development of various tyrosinase inhibitors is actively underway to suppress excessive pigmentation [15,16,17]. Several known tyrosinase inhibitors, such as arbutin, kojic acid, ascorbic acid, and hydroquinone, can be used in cosmetics and pharmaceuticals to treat hyperpigmentation [18,19,20,21,22]. However, these tyrosinase inhibitors have limited use due to serious side effects, such as dermatitis, cytotoxicity, carcinogenicity, and genotoxicity [18,19,20,21,22]. Therefore, there is a need to find other natural compounds that have no undesirable side effects and exhibit the ability to effectively inhibit hyperpigmentation.

Plants are continuously attacked by environmental and chemical pathogens, which degrade the quality and reduce the production rate of various crops [23]. Plants have defense mechanisms to fight pathogens, for which the biosynthesis of secondary metabolites, including phytoalexins, is important. Brassinin belongs to phytoalexins abundant in the family Brassicaceae, including broccoli, cabbage, brussels sprouts, cauliflower, and kohlrabi [24]. Because brassinin is produced by plants in response to environmental stimuli, such as exposure to UV radiation and chemicals, many studies have focused on brassinin synthesis and metabolism in plants. Brassinin is known to have various pharmacological properties, such as antibacterial, anti-obesity, anti-inflammatory, and anti-cancer [25,26,27,28]. In more detail, brassinin exhibits anti-obesity activity by reducing the protein expression levels of adipogenic transcription in 3T3-L1 cells [27]. Brassinin shows anti-inflammatory effects by inhibiting nuclear factor-κB signaling and activating nuclear factor erythroid-2-related factor 2/heme oxygenase-1 in lipopolysaccharide-stimulated murine macrophages RAW264.7 and human monocytes THP-1 [28]. Additionally, brassinin exhibits anti-cancer efficacy by inhibiting the proliferation of various tumor cells and inducing apoptosis [28,29,30]. However, the effect of brassinin on melanogenesis has not yet been tested. In the current study, we tested whether brassinin, which is abundant in various vegetables, shows anti-melanogenic activity using in vitro and in silico analysis.

## 2. Materials and Methods

### 2.1. Materials

Brassinin, 2,4,6-Tris(2-pyridyl)-s-triazine, Iron(II) sulfate heptahydrate, Iron(III) chloride hexahydrate, tyrosinase from mushroom, L-tyrosine, L-DOPA, phenylmethylsulfonyl fluoride, α-melanocyte stimulating hormone (α-MSH), and Triton X-100 were obtained from Sigma-Aldrich (St. Louis, MO, USA). All materials related to cell culture were purchased from Welgene (Daegu, Korea).

### 2.2. Ferric Reducing Antioxidant Power (FRAP) Assay

The reducing power of brassinin was measured with a slight modification of a previously published method [31]. The FRAP working solution was prepared by mixing 300 mM acetate buffer (pH 3.6), 10 mM 2,4,6-Tris(2-pyridyl)-s-triazine, 20 mM ferric chloride, and distilled water in a ratio of 10:1:1:1.2. A total of 6 μL of brassinin and 200 μL of FRAP reagent were added to a 96-well plate and incubated at 37 °C for 4 min. Then, absorbance was measured at 520 nm with a microplate reader (AMR-100, Allsheng, Hangzhou, China). Ferric sulfate was measured at the same concentration as brassinin, and a standard curve was constructed using the average FRAP value versus concentration. This was used to calculate the antioxidant capacity of brassinin.

### 2.3. Cell-Free Mushroom Tyrosinase Activity Assay

Cell-free mushroom tyrosinase activity assay was measured using L-tyrosine as a substrate according to a previously described method [32]. Briefly, 1 μL of brassinin (5, 15, and 30 μM) and 94 μL of an assay mixture buffer (1 mM L-tyrosine solution, 50 mM sodium phosphate buffer (pH 6.5), and distilled water (10:10:9, *v*/*v*/*v*), respectively) were added to the 96-well plates. Then, 5 μL of mushroom tyrosinase (1000 units/mL) was added to each well. Following incubation for 30 min at 37 °C, absorbance was determined at 490 nm using a microplate reader.

### 2.4. Cell Culture

B16F10 (murine melanoma cell line), HaCaT (human keratinocyte cell line), and HS68 (human dermal fibroblast cell line) were purchased from the American Type Culture Collection (ATCC, Rockville, MD, USA). B16F10, HaCaT, and HS68 cells were individually cultured in Dulbecco’s modified Eagle’s medium supplemented with 10% heat-inactivated fetal bovine serum and 1% penicillin-streptomycin in a humidified atmosphere of 5% CO_2_ and 95 % air at 37 °C.

### 2.5. Cell Viability Assay

Cell viability was measured using the MTS assay (CellTiter96^®^ AQueous One Solution Cell Proliferation Assay Kit, Promega, Madison, WI, USA). Briefly, B16F10 (1 × 10^4^ cells/well), HaCaT (5 × 10^3^ cells/well), and HS68 (5 × 10^3^ cells/well) were seeded in 96-well plates. After 24 h, cells were incubated with a fresh medium supplemented with various concentrations of brassinin (1–100 μM) for 24 h. After incubation, 5 μL of MTS reagent was added to each well, and the color was developed at 37 °C for 1 h. The absorbance was determined at 490 nm using a microplate reader.

### 2.6. Measurement of Melanin Content

Measurement of melanin content was performed according to a previously published method [32]. Briefly, B16F10 melanoma cells were incubated in 6-well plates at a density of 5 × 10^3^ cells/well at 37 °C for 24 h with 5% CO_2_ humidified air. Cells were pretreated with 1, 5, and 15 μM of brassinin for 1 h and further stimulated with 500 nM of α-MSH for 6 days. After washing twice with cold PBS, the cells were collected with 100 μL of 1 N NaOH and dissolved at 60° C for 1 h. The 70 μL of cell extracts were placed in a 96-well plate, and absorbance was measured at 490 nm using a microplate reader. The relative melanin content was determined by normalizing melanin values with total protein content.

### 2.7. Cellular Tyrosinase Activity Assay

Intracellular tyrosinase activity was determined by measuring the dopachrome formation of L-DOPA, as described in a previous study [32]. B16F10 melanoma cells were seeded at a density of 5 × 10^3^ cells/well in a 6-well plate. Cells were pretreated with 5 and 15 μM of brassinin for 1 h and further stimulated with 500 nM of α-MSH for 6 days. Cells were washed twice with cold PBS and dissolved with 130 μL of 50 mM sodium phosphate buffer (pH 6.5) containing 1% Triton X-100 and 0.1 mM PMSF. After freezing the cell lysates for 30 min at −80 °C, the supernatant was obtained by centrifugation at 12,000× *g* for 30 min at 4 °C. The 80 µL of the supernatant solution was placed in a 96-well plate, and then 20 μL of 2 mg/mL L-DOPA was added to each well. After incubation at 37 °C for 1 h, the absorbance was measured at 492 nm using a microplate reader. Intracellular tyrosinase activity was determined by normalizing the absorbance with total protein content and calculated as % of control.

### 2.8. Real-Time PCR

The RNA was isolated from B16F10 melanoma cells using the RiboEX^TM^ reagent (GeneAll, Seoul, Korea), and 1 µg of total RNA was used for cDNA synthesis by using a SmartGene Compact cDNA Synthesis Kit (SMART GENE, Daejeon, Korea). Quantitative real-time PCR was performed using the TOPreal^TM^ SYBR Green qPCR PreMIX (Enzynomics, Daejeon, Korea) and QuantStudio^TM^ 1 Real-Time PCR system (Applied Biosystems, Foster City, CA, USA). The expression levels of target genes were calculated using the 2^−ΔΔCT^ method [33].

### 2.9. Immunofluorescence Staining

Immunofluorescence staining was performed according to a previously published method [34]. The cells were fixed with 10% formalin for 1 h at room temperature. After washing twice with DPBS, the cells were treated with 0.5% Triton X-100 for 10 min at room temperature to increase the cell membrane permeability. After blocking for 30 min at room temperature with 0.5% blocking solution, cells were incubated with an anti-microphthalmia-associated transcription factor (MITF) antibody (1:100, sc-515925; Santa Cruz Biotechnology, Santa Cruz, CA, USA) overnight at 4 °C followed by FSD^TM^ 488-conjugated secondary antibody (1:500, RSA1145; BioActs, Incheon, Korea) for 1 h at room temperature. After washing twice with DPBS, the cells were stained with 2 μg/mL 4′6′-diamidino-2-phenylindole dihydrochloride and observed using a fluorescence microscope (LS40, Leamsolution, Gyeonggi-do, Korea).

### 2.10. Docking Simulation

Docking simulation was performed using Auto dock Vina 1.1.2 (free software provided by http://vina.scripps.edu (accessed on 13 October 2021)). Binding affinity was predicted using the 3D structure of a tyrosinase protein derived from *Agaricus bisporus* (PDB ID: 2Y9X). Arbutin and kojic acid are well-known tyrosinase inhibitors. The binding position of arbutin (CID: 440936) and kojic acid (CID: 3840) to tyrosinase was calculated through the binding site of tropolone, and the possibility of brassinin (CID: 3035211) binding to the same position was predicted. A database was searched on Pubchem to obtain the ligand structures of arbutin, kojic acid, and brassinin. Subsequently, LigandScout 4.4 software was used to predict the structural binding potential between tyrosinase and different compounds.

### 2.11. Statistical Analysis

All experimental results were expressed as the mean ± SEM of at least three independent experiments. Significance between groups was conducted with GraphPad Prism 5.0 (GraphPad software, La Jolla, CA, USA) using one-way analysis of variance (ANOVA) according to Tukey’s multiple comparison test.

## 3. Results

### 3.1. Brassinin Is an Antioxidant and Tyrosinase Inhibitor

**Figure 1:**
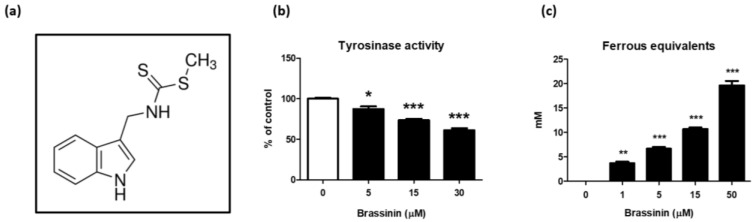
Inhibitory effects of brassinin on mushroom tyrosinase activity and oxidative stress in test tubes. (**a**) The chemical structure of brassinin. (**b**) The tyrosinase inhibitory activity of brassinin was measured in the cell-free system using mushroom tyrosinase as an enzyme and L-tyrosine as a substrate (*n* = 3/group). (**c**) The antioxidant activity of brassinin was determined by FRAP assay in a cell-free system (*n* = 3/group). Results are presented as a mean ± SEM. * p < 0.05, ** p < 0.01, *** p < 0.001 compared with the non-treated control group.

Tyrosinase is a key enzyme responsible for melanogenesis [35]. To investigate whether brassinin (Figure 1a) can be applied to a whitening agent, a test tube-based tyrosinase activity assay was performed using mushroom tyrosinase. Brassinin treatment (5–30 µM) decreased tyrosinase activity in a concentration-dependent manner (Figure 1b). Agents with antioxidative activity, including ascorbic acid, L-cysteine, and 4-hexylresorcinol, can prevent melanin production by binding to the intermediates [36]. We tested whether brassinin exhibits antioxidant activity using FRAP and data showed that brassinin (1–50 µM) concentration-dependently elevates antioxidant capacity (Figure 1c).

### 3.2. Brassinin Did Not Affect Cell Viability in the Range of 1 to 50 µM

**Figure 2:**
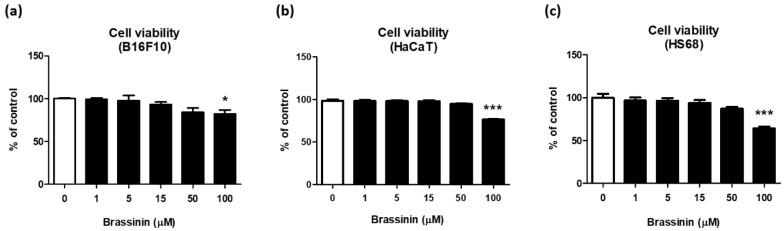
Effects of brassinin on cell viability in B16F10, HaCaT, and HS68 cells. Cell viability was measured in (**a**) B16F10, (**b**) HaCaT, and (**c**) HS68 cells treated with various concentrations of brassinin (1–100 μM) for 24 h (*n* = 4/group). Results are presented as a mean ± SEM. * p < 0.05, *** p < 0.001 compared with the non-treated control group.

We further tested whether brassinin showing antioxidant and tyrosinase-inhibitory activities have the anti-melanogenic capacity in B16F10 cells that are frequently used to find anti-melanogenic compounds. Because safety is the most important issue that should be considered for the application of natural compounds as a cosmetic agent, we performed a cytotoxicity assay using multiple skin cell lines, including HS68 (human fibroblast), HaCaT (human keratinocyte), and B16F10 (mouse melanoma cells). When brassinin was treated in the range of 1–100 µM for 24 h, no cytotoxicity was found between 1 and 50 µM concentration, but slight toxicity was observed at 100 μM in B16F10 (Figure 2a), HaCaT (Figure 2b), and HS68 (Figure 2c) cells. Based on these results, brassinin can be used in subsequent experiments in the concentration range of 1 to 50 μM.

### 3.3. Brassinin Inhibits α-MSH-Induced Intracellular Melanin Accumulation and Tyrosinase Activity in B16F10 Cells

**Figure 3:**
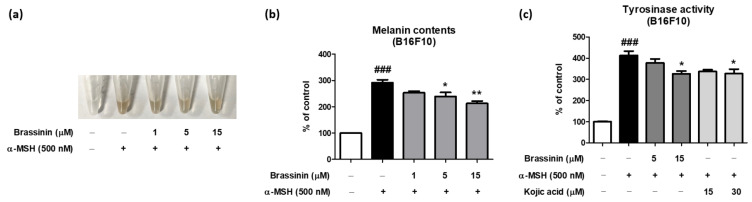
Anti-melanogenesis effects of brassinin in α-MSH-induced B16F10 melanoma cells. (**a**,**b**) Intracellular melanin contents and (**c**) tyrosinase activity were measured in B16F10 cells pretreated with various concentrations of brassinin (1–15 μM) and kojic acid (15, 30 μM) for 1 h and then stimulated with α-MSH (500 nM) for 6 days (*n* = 3/group). Results are presented as a mean ± SEM. *^###^ p* < 0.001 compared with the non-treated control group and * p < 0.05, ** p < 0.01 compared with the α-MSH-treated group.

For a cellular melanogenesis assay, B16F10 melanoma cells were pretreated with brassinin (1–15 µM) for 1 h followed by α-MSH treatment. Then, the cells were cultured for an additional 6 days to stimulate melanin accumulation. As expected, α-MSH treatment markedly elevated cellular melanin content by approximately 180% and the treatment of brassinin decreased it in a dose-dependent manner (Figure 3a,b). Because mushroom tyrosinase activity was reduced by brassinin treatment in a cell-free experiment, we hypothesized that brassinin reduces cellular tyrosinase activity to suppress melanogenesis. When a rough enzyme fraction was isolated from B16F10 cells treated with α-MSH or brassinin and a tyrosinase activity assay was performed, cellular tyrosinase activity was significantly lower in the brassinin-treated group than in the α-MSH group (Figure 3c). These results suggest that brassinin reduces cellular tyrosinase activity to suppress melanogenesis.

### 3.4. Brassinin Inhibits MITF Translocation into the Nucleus and Decreases the mRNA Level of Tyrosinase in α-MSH-Stimulated B16F10 Cells

**Figure 4:**
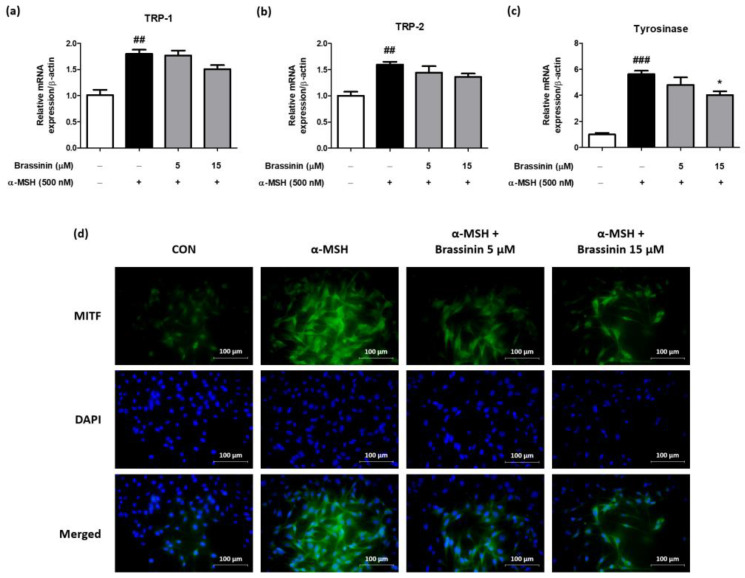
Effects of brassinin on mRNA expression levels of melanogenesis-related genes and MITF translocation. B16F10 melanoma cells were pretreated with brassinin (5, 15 μM) for 1 h then stimulated with α-MSH (500 nM) for 6 days to determine mRNA expression levels of (**a**) TRP-1, (**b**) TRP-2, and (**c**) tyrosinase, respectively (*n* = 3/group). (**d**) MITF was detected using an anti-MITF monoclonal antibody and an FSD^TM^-conjugated secondary antibody. Results are presented as a mean ± SEM. *^##^ p* < 0.01, *^###^ p* < 0.001 compared with the non-treated control group and * p < 0.05 compared with the α-MSH-treated group.

To find a molecular mechanism underlying the brassinin-mediated anti-melanogenesis, we measured mRNA expression levels of genes associated with melanogenesis, including TRP-1, TRP-2, and tyrosinase (Figure 4a–c). α-MSH significantly upregulated TRP-1, TRP-2, and tyrosinase, and brassinin treatment only significantly reduced the mRNA level of tyrosinase among these genes (Figure 4a–c). Since MITF is an important transcription factor for the regulation of tyrosinase, immunofluorescence analysis was performed to evaluate the inhibitory effect of brassinin on MITF translocation into the nucleus. Fluorescent microscopic evaluation showed that the treatment of brassinin inhibited MITF translocation into the nucleus compared with α-MSH-only treated B16F10 melanoma cells (Figure 4d). These results suggest that brassinin inhibits melanin accumulation by reducing the mRNA level of tyrosinase through the inhibition of MITF translocation.

### 3.5. Brassinin May Bind to and Inactivate Tyrosinase

**Figure 5:**
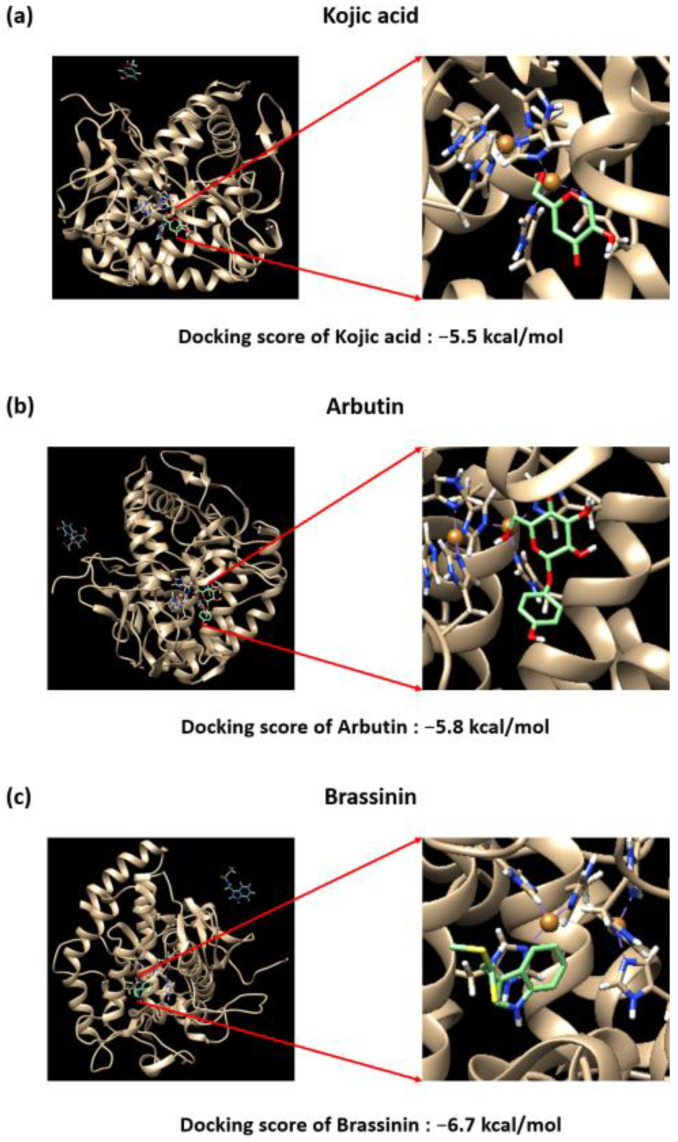
Binding affinity between *Agaricus bisporus* (PDB ID: 2Y9X) and (**a**) kojic acid (CID: 3840), (**b**) arbutin (CID: 440936), or (**c**) brassinin (CID: 3035211). AutoDock Vina software was used to predict the ligand binding capacity of brassinin, which exhibits strong inhibitory activity against tyrosinase.

**Figure 6:**
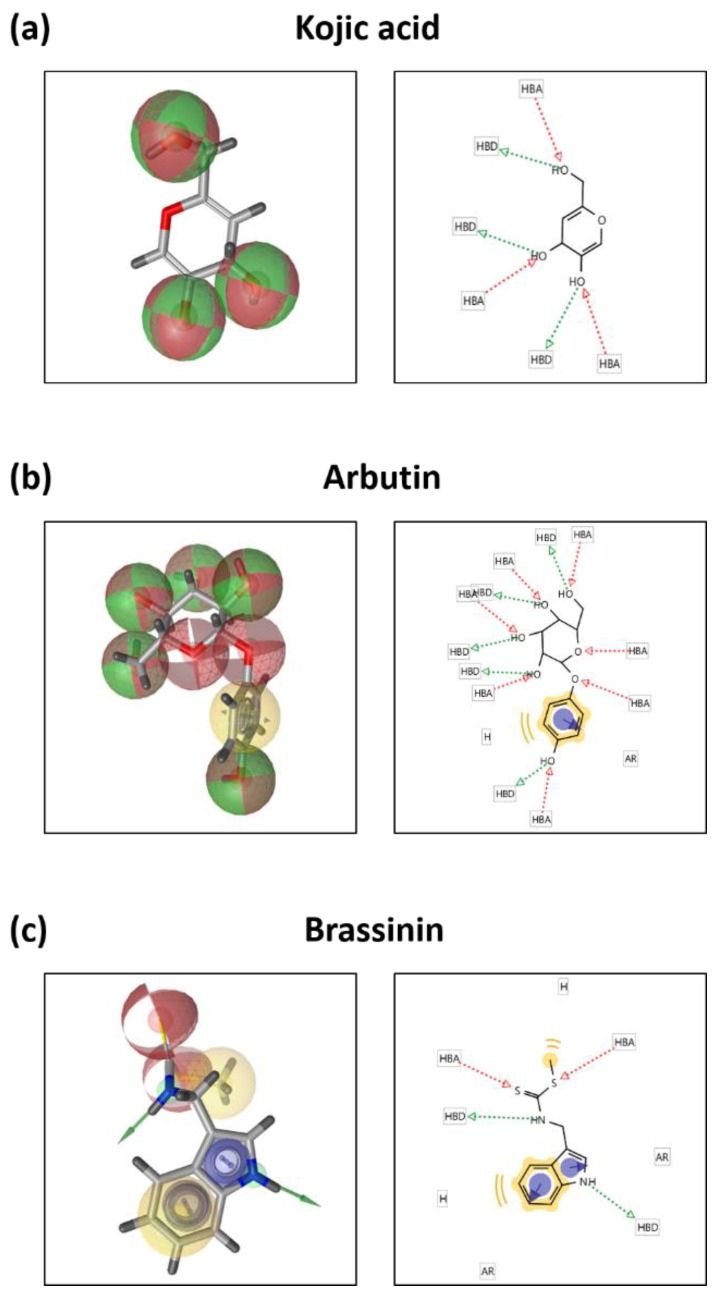
Binding residue analysis of ligands to tyrosinase. Pharmacopoeia analysis of (**a**) kojic acid, (**b**) arbutin, or (**c**) brassinin and tyrosinase was performed using LigandScout 4.4 software.

A protein-ligand docking simulation was performed to predict the binding affinity of brassinin, kojic acid, arbutin, and tyrosinase. We compared the docking scores by predicting the binding strength of brassinin at the site to where the well-known tyrosinase inhibitor tropolone bind. The binding affinity between tyrosinase and kojic acid was –5.5 kcal/mol (Figure 5a), and the binding affinity to arbutin was –5.8 kcal/mol (Figure 5b). Brassinin was –6.7 kcal/mol (Figure 5c), which was higher than that of kojic acid and arbutin. This study confirms that brassinin acts as a strong tyrosinase inhibitor. Binding residue analysis between tyrosinase and compounds as further investigated using Ligand Scout 4.4 software. As predicted by the structure, kojic acid has three hydrogen bond acceptors and three hydrogen bond donors, which may be involved in tyrosinase binding (Figure 6a). Arbutin forms seven hydrogen bond acceptors, five hydrogen bond donors, one aromatic interaction, and one hydrophobic interaction that can be associated with tyrosinase binding (Figure 6b). Analysis of brassinin indicates that it can have two hydrogen bond acceptors and two hydrogen bond donors and can form two aromatic and hydrophobic interactions, which may be associated with the binding affinity (Figure 6c).

## 4. Discussion

Here, we tested whether brassinin affects melanin accumulation in B16F10 cells, and data showed that brassinin decreased α-MSH-induced melanin accumulation in B16F10 cells, presumably by binding to and inactivating cellular tyrosinase and down-regulating tyrosinase expression levels.

Although brassinin is rich in the family Brassicaceae, a few studies investigated the anti-melanogenic activity of their extracts. A study investigated extraction characteristics and tyrosinase inhibitory activity of *Brassica oleracea var. capita* extracts [37]. The maximal tyrosinase inhibition rate of the extracts was approximately 68.9% at the ratio of solvent to sample, EtOH concentration, and extracting temperature of about 24.0 mL/g, 10.5%, and 78.7 °C, respectively. Nevertheless, the study did not provide specific compounds within the extracts of *Brassica oleracea var. capita* for tyrosinase inhibition. Other studies indicate that phytoalexins, including brassinin, can be detected in *Brassica juncea* and *Brassica rapa* in response to environmental or chemical stimulations, including UV radiation, jasmonic acid, fungal spores, CuCl_2_, Dextruxin B, and yeast extract [23]. Because the family Brassicaceae may grow under constant exposure to environmental stimuli, such as heavy metals and UV radiation, some plants in Brassicaceae likely include brassinin in the condition without extra stimulations, although few related studies are available to date.

Melanogenesis is controlled by a variety of signaling pathways in response to intrinsic and extrinsic stimulations, including hormones, UV irradiation, etc. α-MSH elevates intracellular cyclic adenosine monophosphate, resulting in protein kinase A activation through separating catalytic subunits from regulatory subunits. As a result, the catalytic protein kinase A can translocate to the nucleus, phosphorylating and activating cyclic AMP response element binding, which transactivates MITF, an important transcription factor to stimulate the transcription of multiple genes associated with melanogenesis, including tyrosinase [38]. Based on our data, tyrosinase activity was suppressed by brassinin in cell-free experiments. A higher docking score between tyrosinase and brassinin than other positive controls further supports the possibility that brassinin binds to and inactivates tyrosinase. Nevertheless, this is not the sole mechanism underlying brassinin-mediated anti-melanogenic effects because mRNA expression levels of tyrosinase were also significantly reduced in the brassinin-treated group. Thus, we assume that the reduced intracellular tyrosinase activity is derived from both direct bindings to tyrosinase and a decrease in tyrosinase expression by brassinin treatment.

The expression of tyrosinase is mainly regulated by MITF, a transcription factor activated by various intracellular protein kinases, such as extracellular signal-regulated kinases (ERKs), c-Jun N-terminal kinases (JNKs), p38, protein kinase B (Akt), and ribosomal protein S6 kinase (S6K). Based on our data, although translocation of MITF was inhibited, the mRNA levels of ERK1/2, JNK, p38, Akt, and S6K were unchanged by brassinin treatment (Supplementary Figure S1). Previous studies showed that the phosphorylation of ERKs, JNKs, p38, Akt, and S6K upregulated MITF translocation and tyrosinase expression in α-MSH-stimulated B16F10 cells. Therefore, we assume that phosphorylation levels of ERKs, JNKs, p38, Akt, and S6K may be more important for α-MSH-induced melanogenesis. It is necessary to further study whether brassinin affects the phosphorylation levels of ERKs, JNKs, p38, Akt, and S6K in B16F10 cells.

Furthermore, brassinin showed strong antioxidant ability based on FRAP analysis. These results were consistent with other studies that demonstrated the antioxidant activity of brassinin via free radical scavenging assays, such as DPPH, superoxide, peroxyl radicals, and ferric-reducing power [39]. Antioxidants can not only inhibit the initiation of enzymatic browning by reacting with oxygen but also can react with intermediate products to break the chain reaction and prevent melanin formation [40]. Based on these results, we assume that brassinin exerts anti-melanogenic effects, in part, by enhancing antioxidant activity and decreasing intracellular tyrosinase activity.

Brassinin may inactivate tyrosinase, presumably by binding to the active site of tyrosinase based on docking simulation followed by the binding residue analysis. However, it should be noted that docking simulation has limitations, including the lack of confidence in scoring functions to show accurate binding energy because some intermolecular reciprocal action terms are not easy to exactly predict [41]. Although some further experiments suggested above are necessary, our data indicate that brassinin suppressed α-MSH-mediated melanogenesis in B16F10 cells via dual mechanisms, including regulating the activity and expression of tyrosinase.

## 5. Conclusions

Brassinin suppressed α-MSH-mediated melanogenesis at least partly by inhibiting both the activity and mRNA expression levels of tyrosinase. Although further mechanistic and toxicity studies are necessary, the results of this study indicate that brassinin has the potential for use as a depigmenting and cosmeceutical agent.

## Data Availability

Data is contained within the article or supplementary material.

## Supplementary Materials

The following supporting information can be downloaded at: https://www.mdpi.com/article/10.3390/foods12010121/s1, Figure S1: Effects of brassinin on melanogenic upstream signaling pathways in B16F10 melanoma cells.
