# Supplementary material for: Brassinin Abundant in Brassicaceae Suppresses Melanogenesis through Dual Mechanisms of Tyrosinase Inhibition
Source: Foods. 2022 Dec 26;12(1):121. doi: 10.3390/foods12010121 (PMC9818315; doi:10.3390/foods12010121)
Supplement: Supplementary file 1 [file foods-12-00121-s001.zip › foods-2088091-Supplementary.pdf]

Supplementary information

Supplementary Figure S1

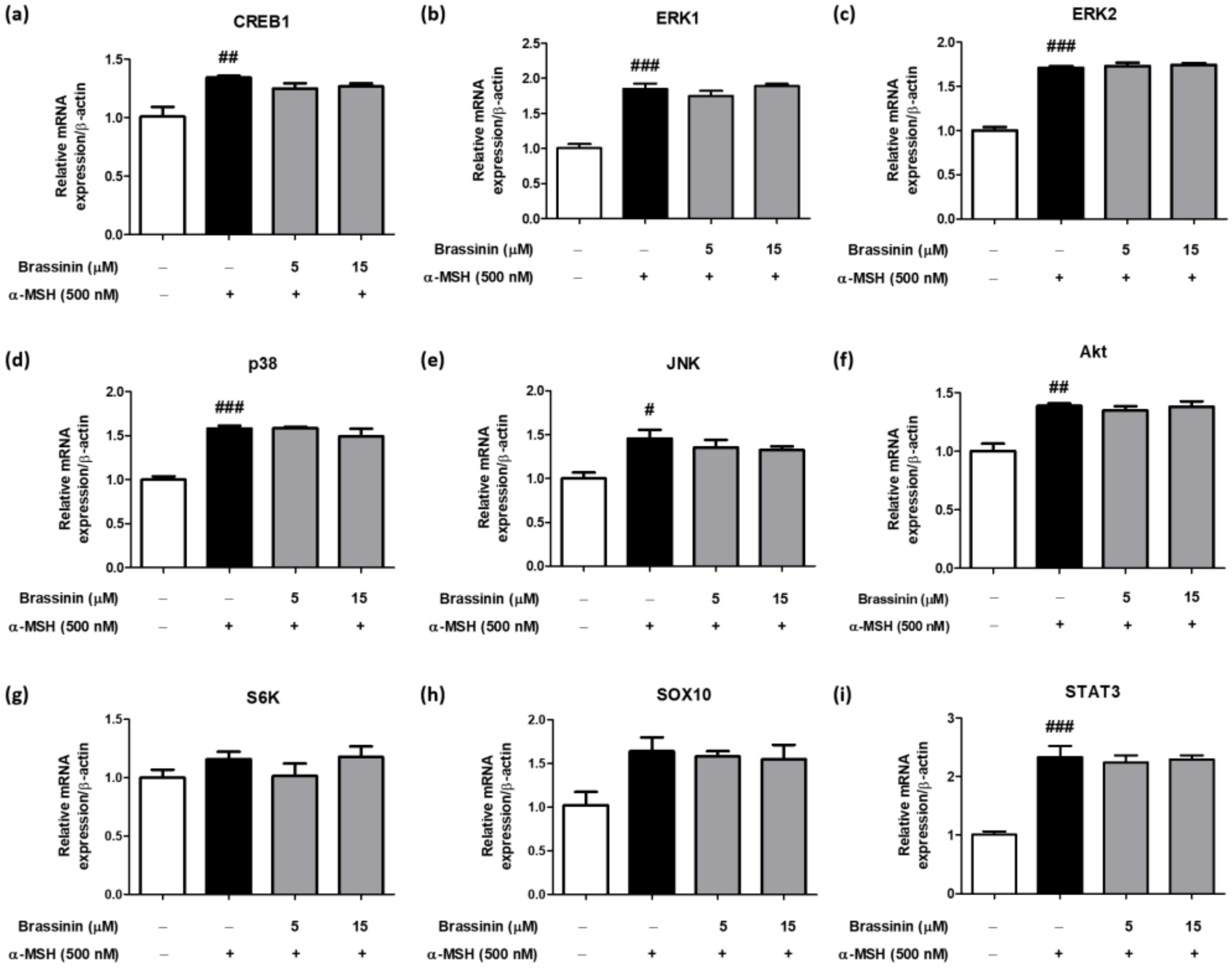

Supplementary Figure S1. Effects of brassinin on melanogenic upstream signaling pathways in B16F10 melanoma cells. B16F10 melanoma cells were pretreated with brassinin (5, 15 μM) for 1 h then stimulated with *α*-MSH (500 nM) for 6 days to determine mRNA expression levels of (**a**) CREB1, (**b**) ERK1, (**c**) ERK2, (**d**) p38, (**e**) JNK, (**f**) Akt, (**g**) S6K, (**h**) SOX10, and (**i**) STAT3, respectively (n = 3/group). Results are presented as a mean ± SEM. # $p < 0.05$, ## $p < 0.01$, ### $p < 0.001$ compared with the non-treated control group.
